# Osteopontin expression correlates with adhesive and metastatic potential in metastasis-inducing DNA-transfected rat mammary cell lines

**DOI:** 10.1038/sj.bjc.6601683

**Published:** 2004-04-27

**Authors:** V E Moye, R Barraclough, C West, P S Rudland

**Affiliations:** 1 School of Biological Sciences, Biosciences Building; 2 Department of Public Health, University of Liverpool, Liverpool L69 7ZB, UK

**Keywords:** osteopontin mRNA, transfected cells, adhesion, rat mammary metastasis

## Abstract

A metastatic phenotype can be induced in benign rat mammary cells (Rama 37 cells) by transfecting them with metastasis-inducing DNAs (Met-DNAs). Stable transfection of Met-DNAs increases the level of the metastasis-associated protein, osteopontin. Randomly picked clonal cell lines have been established from the pool of Rama 37 cells transfected with one metastasis-inducing DNA, C9-Met-DNA. In these cell lines, moderate correlation is observed between the copy number of C9-Met-DNA and their metastatic potential (linear regression coefficient, *R*^2^=0.48). A very close correlation is observed between the cell lines’ metastatic potential *in vivo* and the osteopontin mRNA levels *in vitro* (*R*^2^=0.74), but not with another metastasis-associated protein in this system, S100A4 (*R*^2^=0.21). A close correlation is also observed between osteopontin mRNA levels and the adhesive potential (*R*^2^=0.91) of the cells, but not with their growth rate *in vitro* (*R*^2^=0.03). These observations support the previous suggestion that osteopontin is the direct effector of C9-Met-DNA and that the presence of C9-Met-DNA is necessary, if not sufficient, for the induction of metastasis *in vivo* in this system. Additionally, these results suggest that Rama 37 cells with increased osteopontin mRNA levels become metastatic not through an increased growth rate, but through an increase in cellular adhesiveness.

Breast cancer is one of the leading causes of death from cancer in women (Pisani *et al*, 1999a, 1999b). More specifically, it is the metastasis of the primary breast tumour to distant sites in the body that causes the majority of deaths attributed to breast cancer. Metastasis of breast tumours is frequently life threatening with only 20% of patients diagnosed with metastatic disease surviving after 5 years (American Cancer Society, 1998). One assay for genes that can induce metastasis in breast cells uses the karyotypically stable rat mammary epithelial cell line Rama 37, which is derived from a benign 1, 12 dimethyl benz(a) anthracene-induced mammary tumour as a recipient (Dunnington *et al*, 1983). The Rama 37 cell line yields nonmetastasising tumours when injected into the mammary fat pads of syngeneic rats. However, when this cell line is transfected with certain genes, the primary tumours metastasise to the lungs and lymph nodes. Transfected genes that can induce metastasis in this system include those for S100A4 (Davies *et al*, 1993), osteopontin (Oates *et al*, 1996) and Cutaneous Fatty Acid Binding Protein (Jing *et al*, 2000), but not conventional growth-promoting genes such as Ha-ras (Davies *et al*, 1994) and FGF2 (Davies *et al*, 1996). The products of those genes, which can induce a metastatic phenotype when transfected into Rama 37 cells, have also been shown to occur at higher levels in malignant than in benign, human breast tumours (Jing *et al*, 2000; Platt-Higgins *et al*, 2000; Rudland *et al*, 2002) and for S100A4 and osteopontin are associated with premature death of patients from metastatic breast disease (Platt-Higgins *et al*, 2000; Rudland *et al*, 2002).

In an attempt to identify further regions of the genome that may be involved in metastasis, restriction enzyme-fragmented DNA of the malignant cell line Ca2-83 from a metastatic breast cancer was transfected into the Rama 37 cell line and tested for metastatic capacity in the animal model system (Davies *et al*, 1994). Six fragments of metastasis-inducing DNA (Met-DNA) were subsequently identified which could induce a metastatic phenotype when transfected into the Rama 37 cell line. All Met-DNAs separately induced the expression of osteopontin (Chen *et al*, 1997), in at least one case (C9-Met-DNA) by the removal of an inhibitory transcription factor Tcf-4 from the osteopontin promoter (El-Tanani *et al*, 2001). Although the six Met-DNAs produced different elevated levels of osteopontin mRNA in the resultant pools of transfectants, there was no obvious correlation between these levels and the number of animals bearing metastases, suggesting that osteopontin may be acting indirectly (Chen *et al*, 1997). Moreover, although the mechanism by which osteopontin overexpression induces a metastatic phenotype in the Rama 37 system is unknown, there is evidence to link osteopontin with many of the cellular events that are involved in metastasis, such as avoidance of the immune system, cellular migration (Liaw *et al*, 1995; Singh *et al*, 1995; Tuck *et al*, 2000), invasion (Tuck, 1999) and adhesion (Liaw *et al*, 1994; Barry *et al*, 2000).

In this report, nine clonal cell lines have been established from the original pool of C9-Met-DNA-transfected Rama 37 cells and the molecular and cellular characteristics of each of these clonal cell lines have been investigated. The results now show that osteopontin is the direct effector of metastasis in this system. Additionally, a direct correlation between osteopontin mRNA levels, metastatic potential and cellular adhesion has been shown for the first time, leading to the suggestion that osteopontin induces metastasis in Rama 37 cells, at least in part, by a process reflected in their increased adhesiveness on plastic substrata and not by providing any growth advantage over the untransfected Rama 37 cells.

## MATERIALS AND METHODS

### Cell culture

All cell lines were maintained as described previously (Dunnington *et al*, 1983) in a humidified atmosphere of 90% (v v^−1^) air and 10% (v v^−1^) CO_2_ in routine medium (RM) (Dulbecco's modified Eagle's medium (DMEM) (Gibco BRL, Paisely, Scotland) with 5% (v v^−1^) foetal calf serum (FCS) (Sigma, Dorset, UK), 50 ng ml^−1^ insulin and hydrocortisone). The control cell line Rama 800 was obtained from a transplantable rat mammary tumour (Jamieson and Rudland, 1990). Passaging of cells at a 1 : 8 dilution was carried out at 70–80% confluence using trypsin in EDTA, 0.05% (v v^−1^).

### Establishment and testing of clonal cell lines

Clonal cell lines were established from the pool of Rama 37 cells transfected with C9-Met-DNA by plating approximately 10 000 cells in 9 cm diameter dishes. After 1 week, colonies derived from single cells were harvested by placing over the colony a 4 mm diameter disc of Whatman filter paper soaked in trypsin 0.05% (v v^−1^) in EDTA and incubating for 10 min at 37°C. The discs of paper with the cells attached were subsequently placed in one well of a 24-well plate in cloning medium (CM) (45% (v v^−1^) DMEM (Gibco BRL), 10% (v v^−1^) FCS (Sigma), 45% (v v^−1^) RPMI (Gibco BRL), 50 ng ml^−1^ hydrocortisone and insulin). When there were sufficient cells to be plated into 4, 9 cm diameter Petri dishes, they were transferred and maintained in RM. Plating efficiency was determined by plating the cell lines at a known density (20 000–30 000 cells per dish) in conditioned media generated from the RM of cells that had been growing exponentially in a 9 cm diameter plate for 3 days. After plating and incubation for 15 min at 37°C to allow cells to adhere to the plate, the cells were removed from the plate with trypsin/EDTA solution and counted in a Coulter counter. Plating efficiency was calculated as the percentage of cells that had adhered after 15 min. Three independent experiments were carried out in triplicate and error bars represent the standard deviation of the three separate experiments. Cell growth assays were carried out by plating a known density of cells (5–10 000 per well) in 24-well plates. Every day for 6 days, cells in a set of triplicate wells were completely removed by trypsinisation and counted in a Coulter counter. Growth curves were plotted and the gradients of the curves were obtained using linear regression analysis. Growth rates were subsequently represented as a percentage of that for the Rama 37 cell line. Experiments were carried out three times in triplicate and the error bars represent the standard deviation of three separate experiments.

### Assay for metastasis

Metastasis assays were carried out as described previously (Dunnington *et al*, 1983). Cells (2 × 10^6^) to be assayed, suspended in 0.2 ml phosphate-buffered saline (PBS), were injected into the right inguinal mammary fat pads of approximately 20 female Furth Wistar rats of 6–10 weeks of age. Animals were observed every 3 days and killed after 3 months. The primary tumour and lungs of each animal were then fixed in Methacarn (60% (v v^−1^) methanol, 10% (v v^−1^) acetic acid and 30% (v v^−1^) Inhibisol) and embedded in paraffin wax. Sections of 5 *μ*m were then cut and stained with haematoxylin and eosin. Analysis of the stained lung sections allowed individual animals to be scored positive or negative for lung metastasis by two observers. Metastatic potential of the cell lines was expressed as the percentage of tumour-bearing animals exhibiting lung metastases. Animals were maintained under UK Home Office Project Licence no. 40/1515 to Professor PS Rudland in accordance with the guidelines set down by the United Kingdom Coordinating Committee for Cancer Research.

### Northern and Southern blotting

DNA and RNA from the cell lines were isolated by ultracentrifugation through caesium chloride gradients of guanidine isothiocyanate cell extracts, as described previously (Coombs *et al*, 1990; Anandappa *et al*, 1994). Southern transfer was carried out as described in Sambrook *et al* (1989). Ten *μ*g of *Eco*R1-digested genomic DNA was resolved on a 0.8% (w v^−1^) agarose gel and blotted overnight on to Hybond-N membrane (Amersham Pharmacia, Buckinghamshire, UK) in 20 × SSC. DNA was fixed on the membrane by exposure to UV light. RNA for Northern blotting was resolved on a 0.8% (w v^−1^) formaldehyde agarose gel and blotted onto Hybond-N membrane (Amersham Pharmacia) in 10 × SSC. RNA was fixed onto the membrane by exposure to UV light. Probes radioactively labelled with *α*-[^32^P]dCTP (ICN, Hampshire, UK) (specific activity >1 × 10^8^ d.p.m *μ*g^−1^ DNA) were generated for Northern and Southern blotting using a random primed labelling kit (Roche Molecular Chemicals, Hertfordshire, UK). The 1500 bp osteopontin cDNA probe was obtained by digestion of the pBKCMV-OPN plasmid (Oates *et al*, 1996) with restriction enzymes *Xba*1 and *Kpn*1. The 300 bp S100A4 cDNA was a PCR product of full-length rat S100A4 cDNA obtained from Dr I Hickson (University of Liverpool, UK). The 1000 bp C9-Met-DNA probe was obtained by digestion of the pKS-C9-Met-DNA plasmid (Chen *et al*, 1997) with *Hind*III. Hybridisation for Northern and Southern blots was carried out at 42°C in formamide buffer (5 × SSC, 50% (v v^−1^) formamide, 1 × Denhardt's solution (Sambrook *et al*, 1989), 5% (w v^−1^) dextran sulphate and 3 *μ*g ml^−1^ sonicated salmon sperm DNA). After washing the membranes consecutively with: (1) 2 × SSC, 0.1% (w v^−1^) sodium dodecyl sulphate (SDS); (2) 0.2 × SSC, 0.1% (w v^−1^) SDS; and (3) 0.1 × SSC, 0.1% (w v^−1^) SDS, hybridisation signals were visualised by exposing the wrapped membrane to autoradiographic film for 6–72 h with an intensifying screen. Hybridisation signals were quantified using a Model XL 77CECCD video camera and MacIntosh IMAGE and PHOTO computer packages (NIH, Bethesda, MD, USA). C9-Met-DNA copy number was determined by comparing the intensity of C9-Met-DNA hybridised bands from the Rama cells to plasmid copy number controls on the same membrane. Although the amount of plasmid DNA and cellular DNA loaded onto the gel was different, comparison of relative intensities of hybridised bands of plasmid DNA and cellular DNA give a reliable estimate of copy number (Sambrook *et al*, 1989), as described previously (Davies *et al*, 1994; Oates *et al*, 1996; Chen *et al*, 1997). Equal loading of cellular DNA of the transformant and parental cell lines was indicated by equal sample intensity on the gel following electrophoresis and staining with ethidium bromide. There were no higher molecular weight bands hybridising to the OPN cDNA probe, indicating that digestion of genomic DNA by *Eco*R1 was complete in all cases. Osteopontin and S100A4 mRNA levels were normalised for differences in loading/transfer by dividing the intensity of the cDNA hybridised band by the intensity of the ethidium bromide-stained 28S ribosomal RNA band, rather than the intensity of an actin cDNA-hybridised band which could have possibly had its expression changed by C9-Met-DNA.

### Statistical analysis

The Student's *t*-test and linear regression analysis to determine correlation coefficients were carried out using the Microsoft EXCEL package. The significance of the latter fit to a straight line was calculated, *P* values of less than 0.05 were considered statistically significant.

## RESULTS

### Metastatic potential of clonal cell lines

Nine clonal cell lines were established from the original pool of Rama 37 cells transfected with C9-Met-DNA, they were termed R37C9VM followed by clone numbers. Within the set of clonal cell lines, various cellular morphologies were observed, ranging from tightly clustered cuboidal to a more elongated morphology (Table 1

**Table 1:** Morphology in culture and incidence of tumours and metastases *in vivo* of C9-Met-DNA transfectants

**Cell line**	**Morphology in culture**	**No. of rats**	**No. of tumour bearing rats^a^**	**No. of tumour bearing rats with metastases^b^**	**Percent metastasis^c^**
R37	Cuboidal	20^d^	16^d^	0^d^	0^d^
R37C9VM3	Cuboidal with pseudopodia	21	21	15^e^	71^e^
R37C9VM6	Cuboidal, tightly clustered	20	0	0	0
R37C9VM8	Pseudocuboidal^f^	22	22	7^e^	32^e^
R37C9VM13	Pseudocuboidal^f^	21	21	11^e^	52^e^
R37C9VM14	Pseudocuboidal^f^ with pseudopodia	18	18	12^e^	67^e^
R37C9VM16	Cuboidal, tightly clustered	20	20	8^e^	40^e^
R37C9VM18	Elongated with pseudopodia	18	18	8^e^	44^e^
R37C9VM25	Elongated	21	21	13^e^	62^e^
R37C9VM29	Cuboidal, very tightly clustered.	18	18	6^e^	33^e^
R37C9Pooled	Mixed	21^d^	21^e^	5^e^	24^e^

a Assayed after 3 months.

b Determined histologically.

c No.of tumour-bearing rats with metastases/no. of tumour-bearing rats × 100.

d Data from Chen *et al*, 1997.

e Significantly greater than Rama 37 (*P*<0.05; Fisher's exact test).

f Apparently cuboidal in morphology caused by over- and underlapping of elongated cellular processes (Warburton *et al*, 1981; Rudland *et al*, 1989).

). Eight of the nine clonal cell lines produced primary tumours in all animals. The exception was the cell line R37C9VM6 which failed to produce primary tumours in any animals. Of the cell lines that produced primary tumours, lung metastases were detected in 32–71% of the tumour-bearing animals (Table 1). This range compares with the 24% lung metastases reported for the pool of Rama 37 cells transfected with C9-Met-DNA and with no metastases from the Rama 37 cell line (Oates *et al*, 1996; Chen *et al*, 1997) There was no correlation between cellular morphology and percent metastasis (Table 1).

### Osteopontin and S100A4 mRNA levels

Northern blotting showed that six of the nine clonal cell lines had significantly higher levels of the correct size, 1.6 kb, osteopontin mRNA than the Rama 37 cell line, with the increase in levels ranging from 2.8- to 11.1-fold (Student's *t*-test, *P*⩽0.046) (Table 2

**Table 2:** Relative levels of osteopontin and S100A4 mRNA and C9-Met-DNA copy number in the C9-Met-DNA transfectants

**Cell line**	**OPN^a^**	**mRNAS100A4^a^**	**mRNAC9-Met-DNA copy number^b^**
R37	1^c^ (±0.13)	1 (±0.23)	0
R37C9 VM3	11.1 (±0.55)^c^^,^^d^	1.6 (±0.15)	147 (±17)^c^^,^^d^
R37 C9 VM6	0.5 (±0.18)^c^	0.8 (±0.47)	0
R37 C9 VM8	1.4 (±0.14)^c^	1.2 (±0.19)	169 (±8)^c^^,^^d^
R37 C9 VM13	5.5 (±0.20)^c^^,^^d^	1.6 (±0.16)	133 (±10)^c^^,^^d^
R37 C9 VM14	8.0 (±0.5)^c^^,^^d^	0.6 (±0.38)	95 (±6)^d^
R37 C9 VM16	2.8 (±0.16)^c^^,^^d^	1.5 (±0.15)	8 (±3)^d^
R37 C9 VM18	4.5 (±0.24)^d^	0.8 (±0.05)	83 (±5)^d^
R37 C9 VM25	9.3 (±1.1)^c^^,^^d^	2.2 (±0.43)	145 (±17)^c^^,^^d^
R37 C9 VM29	0.7 (±0.21)^c^	1.3 (±0.34)	3 (±0.3)
R37 C9Pooled	4.3 (±0.28)^d^	0.8 (±0.14)	50^d^^,^^e^

a Relative mRNA levels determined by Northern blotting.

b C9-Met-DNA copy number determined by Southern blotting

c Significantly different from C9-Met-DNA pooled transfectants (*P*<0.05; Student’s t-test).

d Significantly different from Rama 37 (*P*<0.05; Student’s t-test).

e eData from Chen et al (1997). Values in brackets represent the mean±standard deviation of three separate experiments.

) (Figure 1A, C

**Figure 1:**
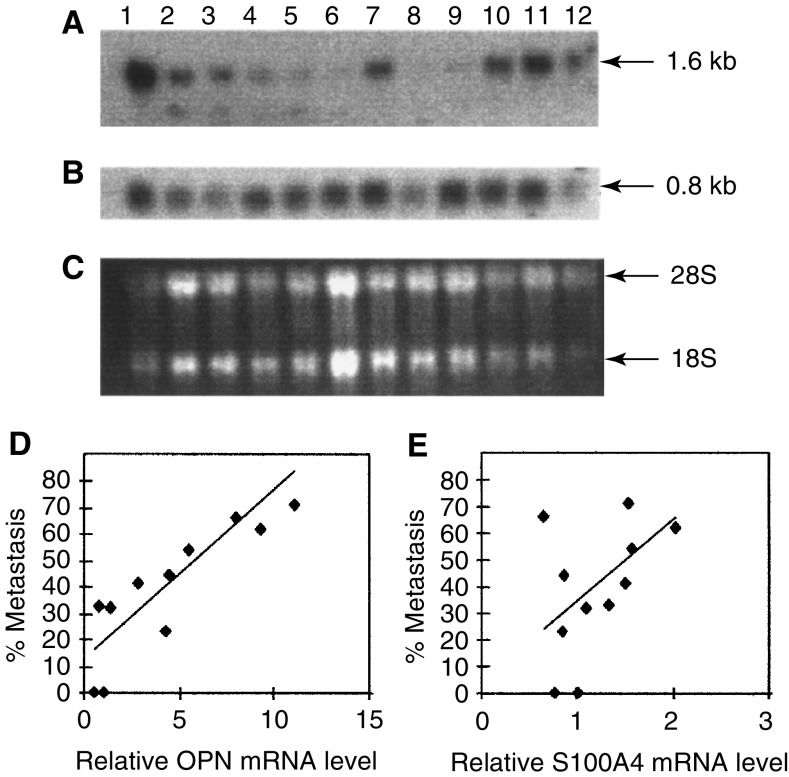
An example of a Northern blot for OPN and S100A4 mRNA levels in the clonal cell lines. The following samples were electrophoresed through 0.8% (w v^−1^) formaldehyde agarose gels and blotted onto Hybond-N membrane: Rama 800 (lane 1), R37C9Pooled (lane 2), R37C9VM13 (lane 3), R37C9VM16 (lane 4), R37C9VM8 (lane 5), R37C9VM6 (lane 6), R37C9VM25 (lane 7), Rama 37 (lane 8), R37C9VM29 (lane 9), R37C9VM14 (lane 10), R37C9VM3 (lane 11), R37C9VM18 (lane 12). In panel (**A**) the membrane was incubated with *α*^32^P-labelled OPN cDNA and subjected to autoradiography. In panel (**B**) the membrane was incubated with *α*^32^P-labelled S100A4 cDNA and subjected to autoradiography. In panel (**C**) the ethidium bromide-stained 18S and 28S ribosomal RNA bands on the membrane before hybridisation were photographed. The 1.6 kb OPN mRNA, 0.8 kb S100A4 mRNA and 18S and 28S ribosomal RNAs are shown. (**D**) Graph of OPN mRNA levels of the cloned cell lines in culture relative to that in Rama 37 cells (Relative OPN mRNA level) plotted against their metastatic potential *in vivo* (% Metastasis) (Materials and Methods). (**E**) S100A4 mRNA levels of the cloned cell lines in culture relative to that in Rama 37 cells (Relative S100A4 mRNA level) plotted against their metastatic potential *in vivo* (% Metastasis) (Materials and Methods). (**D**,**E**) Contain results for 9 C9-Met-DNA-transfected Rama 37 cell lines plus the uncloned C9-Met-DNA-transfected cells and the parental Rama 37 cells.

). Osteopontin mRNA levels were corrected for differences in loading/transfer by normalisation to the 28S ribosomal RNA (Materials and Methods). The remaining three cell lines R37C9VM6, R37C9VM8 and R37C9VM29 failed to exhibit significantly elevated levels compared with the Rama 37 cell line. Furthermore, with the exception of R37C9VM18, all the clonal cell lines were shown to be significantly different from the C9-Met-DNA transfectant pool of Rama 37 cells (*P*<0.05) (Table 2). When the metastatic potential *in vivo* and the relative osteopontin mRNA levels in the cultured cell lines were plotted against each other, there was a significant linear relationship between them (linear regression analysis: *R*^2^=0.74, *P*=0.0007) (Figure 1D). The mRNA levels of another metastasis-inducing gene in this system, S100A4, failed to show significant differences for either the clonal cell lines or the pooled transfectants in comparison with that of the Rama 37 cell line (*P*>0.05) (Table 2) (Figure 1B, C). Furthermore, when the metastatic potential of the cell lines *in vivo* was plotted against S100A4 mRNA levels, there was no simple linear relationship between the two variables (linear regression analysis: *R*^2^=0.21, *P*=0.16) (Figure 1E). There was also no correlation between the levels of osteopontin mRNA and S100A4 mRNA (Table 3

**Table 3:** Pairwise comparison of the cellular and molecular characteristics of transfectants

**Pairwise comparison^a^**	***R*^2^^,^^b^**	***P*^c^**
OPN mRNA level/plating efficiency	0.91	5.9 × 10^−6^^d^
OPN mRNA level/growth potential	0.03	0.63
OPN mRNA level/C9-Met-DNA copy no.	0.43	0.03^d^
OPN mRNA level/metastatic potential	0.74	0.0007^d^
OPN mRNA level/S100A4 mRNA level	0.16	0.23
S100A4 mRNA level/plating efficiency	0.21	0.16
S100A4 mRNA level/growth potential	0.01	0.76
S100A4 mRNA level/metastatic potential	0.21	0.16
S100A4 mRNA level/C9-Met-DNA copy no.	0.16	0.22
C9-Met-DNA copy no./metastatic potential	0.48	0.02^d^
Plating efficiency/metastatic potential	0.73	0.0008^d^
Growth potential/metastatic potential	0.2	0.17

a No. of cell lines tested by linear regression analysis is 11: nine clonal cell lines of the C9-Met-DNA-transfected cells plus the uncloned C9-Met-DNA-transfected Rama 37 cells and the parental Rama 37 cells.

b Linear regression coefficient (*R*^2^

c Probability (*P*) that population lies on a straight line.

d Significant at a 5% level.

). The control cell line from a transplantable metastatic tumour, Rama 800 expressed both mRNAs at the correct size (Figure 1A, B).

### C9-Met-DNA copy number

**Figure 2:**
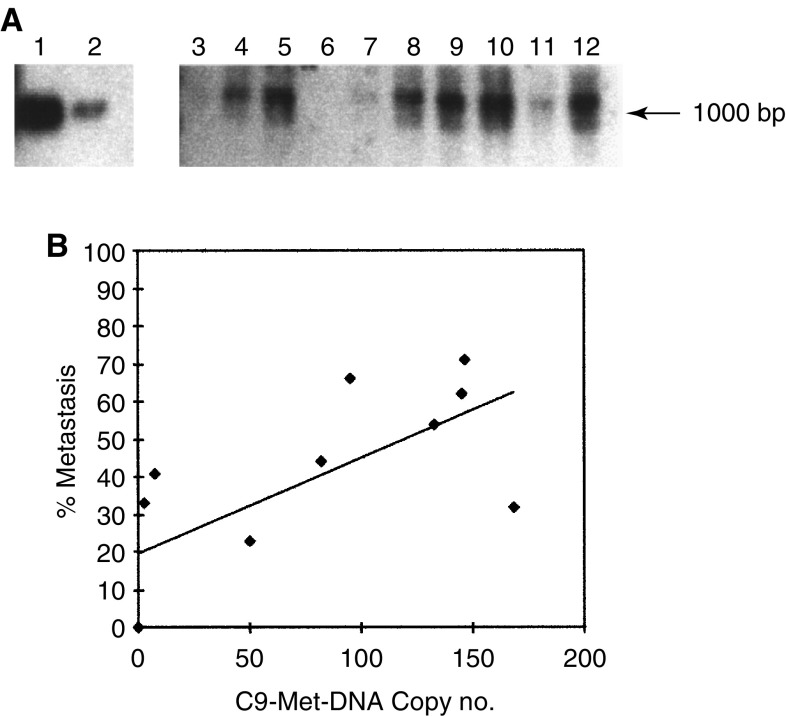
An example of Southern hybridisation of C9-Met-DNA to DNA obtained from the clonal cell lines. (**A**) DNA from each of the cell lines was digested with *Eco*R1, electrophoresed on agarose gels, and blotted onto Hybond-N membrane. The samples loaded on the gel were 500 copies C9-Met-DNA (lane 1), 50 copies C9-Met-DNA (lane 2), Rama 37 DNA (lane 3), R37C9VM18 (lane 4), R37C9VM13 (lane 5), R37C9VM6 (lane 6), R37C9VM29 (lane 7), R37C9VM14 (lane 8), R37C9VM3 (lane 9), R37C9VM8 (lane 10), R37C9VM16 (lane 11) and R37C9VM25 (lane 12). The membrane was incubated with *α*^32^P-labelled C9-Met-DNA under hybridising conditions and subjected to autoradiography. The position of the authentic C9-Met-DNA is shown by the arrow. (**B**) Graph of C9-Met-DNA copy number (copy no) of the cell lines plotted against their metastatic potential *in vivo* (% Metastasis) (Materials and Methods). Results for the nine cloned C9-Met-DNA-transfected cell lines plus Rama 37 parental cells are shown.

The number of copies of C9-Met-DNA integrated into the genome of each of the cell lines was assessed by Southern blotting. Six of the clonal cell lines from the C9-Met-DNA transfectants possessed approximately 80–170 copies of C9-Met-DNA fragments per haploid genome (R37C9VM3, R37C9VM8, R37C9VM13, R37C9VM14, R37C9VM18 and R37C9VM25) (Table 2) (Figure 2A

). The remaining clonal cell lines possessed much lower C9-Met-DNA copy numbers of 0–10 (Table 2) (Figure 2A). Seven of the nine clonal cell lines had significantly higher numbers of C9-Met-DNA copies than the Rama 37 cell line (Student's *t*-test; *P*<0.05) (Table 2). The remaining two cell lines R37VM6 and R37C9VM29 did not have significantly higher levels. Moreover, the clonal cell lines R37C9VM3, VM8, VM13 and VM25 possessed significantly different levels of C9-Met-DNA compared to the C9-Met-DNA transfectant pool of Rama 37 cells (Student's *t*-test; *P*<0.05) (Table 2). When the number of integrated copies of C9-Met-DNA was plotted against the metastatic potential, there was a moderate but significant correlation (linear regression analysis: *R*^2^=0.48, *P*=0.02) (Figure 2B) (Table 3). There was also a moderate but significant correlation between the C9-Met-DNA copy number and the level of OPN mRNA (linear regression analysis: *R*^2^=0.43, *P*=0.03) but not with levels of S100A4 mRNA (linear regression analysis: *R*^2^=0.16, *P*=0.22) (Table 3).

### Growth rates

Growth curves were constructed for each cell line in order to determine their average growth rate. All the cell lines that had been transfected previously with C9-Met-DNA showed a statistically significant reduction in growth rate over that obtained for the untransfected Rama 37 cells (Student's *t*-test; *P*<0.012). When the relative growth rates were plotted against their metastatic potential, there was no statistically significant correlation between the two variables (linear regression analysis: *R*^2^=0.2, *P*=0.17) (Table 3). There was also no correlation of growth rate with levels of osteopontin mRNA (*R*^2^=0.03, *P*=0.63) or of S100A4 mRNA (*R*^2^=0.01, *P*=0.76) (Table 3).

### Adhesive potential

**Figure 3:**
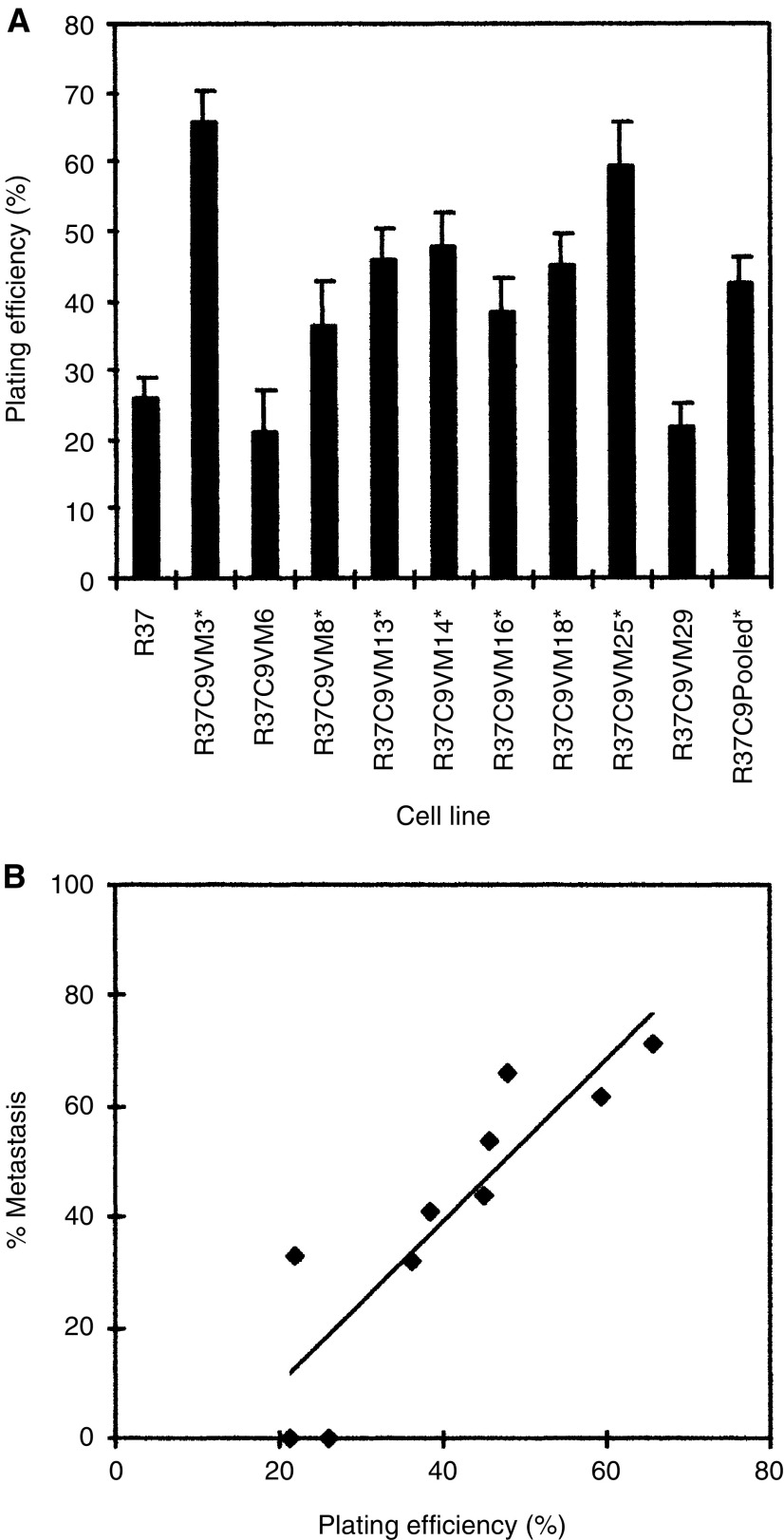
(**A**) Adhesive potential of the transfectant cell lines and the pool of Rama 37 cells transfected with C9-Met-DNA. Plating efficiency defined as percentage of cells attached to plastic substratum after 15 min incubation in self-conditioned medium at 37°C is shown for the parental Rama 37 (R37), the C9-Met-DNA-transfected cell lines (R37C9VM3 etc) and the C9-Met-DNA pooled transfectants (R37C9Pooled). ^*^ Statistically different plating efficiency from the Rama 37 cell line (*P*<0.05; Student's *t*-test). (**B**) Graph of the mean plating efficiency of the cell lines plotted against their metastatic potential *in vivo* (% Metastasis) (Materials and Methods).

Adhesive potential of the C9-Met-DNA transfectants was determined from their plating efficiency over a 15-min period in conditioned medium exposed for 3 days to the growing transfectants (Materials and Methods). Seven of the nine C9-Met-DNA transfectants possessed a statistically increased plating efficiency over that of the untransfected Rama 37 cells (Student's *t*-test; *P*<0.002) (Figure 3A

). The remaining two cell lines, R37C9VM6 and R37C9VM29 failed to show a significant increase. When the plating efficiency was plotted against the metastatic potential, there was a statistically significant linear correlation between these two variables (linear regression analysis: *R*^2^=0.73, *P*=0.0008) (Figure 3B) (Table 3). There was also a highly significant correlation of adhesive potential with levels of OPN mRNA (*R*^2^=0.91, *P*=6 × 10^−6^), but not with levels of S100A4 mRNA (*R*^2^=0.21, *P*=0.16) (Table 3). To test whether the conditioned medium or the cells were causing the increase in adhesiveness of the OPN-expressing transfectants, all the transfectants were retested for adhesive potential in fresh routine medium not exposed to cells. There was no significant difference in plating efficiency for any of the transformant or parental cell lines when tested in routine or conditioned media (Students’ *t*-test, *P*>0.05). Furthermore, under these conditions, there was also a statistically significant linear correlation between plating efficiency and metastatic potential (linear regression analysis, *R*^2^=0.78, *P*=0.00031), and plating efficiency and levels of OPN mRNA (*R*^2^=0.93, *P*=2 × 10^−6^) (not shown); the levels of correlation were similar to those observed earlier when transformant and parental cell lines were tested in their conditioned media rather than in fresh, unconditioned media.

## DISCUSSION

To assess the association of levels of osteopontin mRNA with metastasis and other molecular and cellular characteristics, a series of single-cell-cloned cell lines has been isolated from the original pool of C9-Met-DNA Rama 37 transfectants described by Chen *et al* (1997). That the C9-Met-DNA-transfected clonal cell lines possess a range of morphologies in culture and show different metastatic potentials *in vivo* suggests that the pool of C9-Met-DNA-transfected Rama 37 cells is a mixed population of cell types. This suggestion may explain why a linear correlation between the osteopontin mRNA levels and metastatic potential of the various pooled transfectants produced from the other Met-DNAs (C6-Met-DNA, etc.), although significantly increased, was not observed (Chen *et al*, 1997). For example, the Rama 37 cells transfected with C6-Met-DNA induced a relatively high level of metastasis (50%), but the increase in osteopontin mRNA was relatively modest (1.6-fold), whereas Rama 37 cells transfected with C9-Met-DNA induced a lower level of metastasis (23%), but showed a relatively high increase in osteopontin mRNA (4.4-fold).

Previously, osteopontin mRNA levels have been suggested to be the effector of all the Met-DNA fragments, including C9-Met-DNA in the induction of metastasis (Oates *et al*, 1996; Chen *et al*, 1997). Furthermore, the levels of osteopontin mRNA have been shown to reflect the levels of osteopontin protein in Met-DNA-transfected Rama 37 cells (Oates *et al*, 1996; Chen *et al*, 1997; El-Tanani *et al*, 2001). Now the dose-dependent relationship observed between osteopontin mRNA levels in culture and metastatic potential *in vivo* for the clonal cell lines provides strong evidence that, in the case of these C9-Met-DNA-transfected clonal cell lines, metastatic potential *in vivo* is linearly correlated with the osteopontin mRNA levels found in the cultured cells. Hence, it is highly probable that increases in the relative amounts of osteopontin protein produced by the tumour cells is the final effector of at least C9-Met-DNA. Moreover, another gene known to induce metastasis in Rama 37 cells, that for S100A4 (Davies *et al*, 1993), does not have significantly higher levels of mRNA in the C9-Met-DNA clonal cell lines than in the untransfected Rama 37 cell line. Thus, it is highly likely that increased osteopontin mRNA levels (and hence protein) are the direct cause of the elevated metastatic potential in the C9-Met-DNA-transfected Rama 37 cells. This conclusion is substantiated by experiments that show overexpression of osteopontin by stable transfection of an expression vector in Rama 37 cells induces a metastatic phenotype (Oates *et al*, 1996). Furthermore, the association of osteopontin overexpression both with tumour progression and with metastasis has been documented in other *in vivo* animal models (Craig *et al*, 1990; Gardner *et al*, 1994; Feng *et al*, 1995; Behrend *et al*, 1995) and in human studies (Singh *et al*, 1997; Tuck *et al*, 1997; Rudland *et al*, 2002).

Both the loss and gain of cell–cell adhesion is important for metastasis. For example, in the early stages of metastasis, the cell adhesion molecule E-cadherin is often lost, allowing cells to escape from the primary tumour (Christofori and Semb, 1999). However, at more advanced stages of malignancy, E-cadherin appears to be re-expressed (Bukholm *et al*, 2000). In this report, osteopontin mRNA levels are linearly correlated with cellular adhesive potential in this set of clonal C9-Met-DNA-transfected Rama 37 cell lines. This result suggests that the mechanism by which the increased osteopontin levels cause increased metastatic potential in these cells is by increasing cellular adhesiveness, at least in part, although other mechanisms cannot be excluded. Since there was no difference in the plating efficiency when transformant and parental cell lines were tested in their conditioned or fresh unconditioned media, the increase in adhesiveness of the transfectants arises almost exclusively from the cells themselves and not from material secreted into the conditioned medium. Increases in cellular adhesion could allow attachment of the tumour cells to vessel walls before extravasation and migration to their chosen site of metastasis. This hypothesis is supported by the evidence that osteopontin has also been shown to mediate cellular adhesion of several different types of cells including human breast cancer cells, transformed NIH 3T3 cells and erythroleukaemic cells (Liaw *et al* 1994; Barry *et al*, 2000).

During the process of metastasis, intercellular contact mediated through the cell adhesion molecule E-cadherin appears to prevent apoptosis (Kantak and Kramer, 1998; Day *et al*, 1999). There is growing evidence to suggest that osteopontin may also inhibit apoptosis during metastasis. The inhibition of apoptosis by OPN has been shown in a variety of cell types including human breast cancer cells and endothelial cells (Lopez *et al*, 1996; Khan *et al*, 2002). Additionally, osteopontin-deficient vascular smooth muscle cells show increased apoptosis and decreased adherence. This not only provides further evidence for the role of osteopontin in cellular adherence but also in the inhibition of apoptosis (Weintraub *et al*, 2000). The mechanism by which osteopontin inhibits apoptosis is thought to be via multiple ligand receptor interactions in response to various proapoptotic signals (Denhardt *et al*, 2001). Therefore, osteopontin-mediated adhesion of the tumour cells could be operating in the same manner as re-expressed E-cadherin. Initially apoptosis is inhibited, allowing the tumour cells in the circulation to survive and then through increasing cell–cell adhesion, the tumour cells become attached to blood vessel walls ready for extravasation.

Other properties that can be affected by an increase in osteopontin expression include an increase in matrix metalloproteinase expression (Teti *et al*, 1998), an increase in cellular migration (Liaw *et al*, 1994; Tuck *et al*, 2000) and a protective capacity of tumour cells from macrophage-mediated nitric oxide attack (Denhardt and Chambers, 1994; Feng *et al*, 1995). These potential changes and others have not been assayed in the present system, and it is possible that all, or a combination of properties, not just increased cellular adhesion are responsible for the cell lines with high levels of osteopontin mRNA becoming metastatic *in vivo.* However, the results clearly demonstrate that the C9-Met-DNA-transfected cells have not simply gained a selective growth advantage over untransfected Rama 37 cells, since the growth rates of the clonal cell lines *in vitro* do not correlate separately with either metastatic potential or osteopontin mRNA levels. Moreover, the benign untransfected Rama 37 cell line possesses a significantly faster growth rate *in vitro* than the majority of the clonal cell lines and the latent period before the tumours appear *in vivo* is similar in the parental Rama 37 cell line and in the C9-Met-DNA-transfected clonal cell lines, both results mitigating against growth advantage being the cause of metastasis in this system.

That there is only a moderately significant linear relationship between metastatic potential and C9-Met-DNA copy number suggests that, although the presence of C9-Met-DNA is essential, it may not necessarily be sufficient for the transfected Rama 37 cell to become metastatic *in vivo*. Digestion of genomic DNA by *Eco*R1 was complete in all cases, as described in Materials and Methods, so eliminating the possibility of anomalous numbers of integrated copies of transfected OPN cDNA being recorded. C9-Met-DNA has been postulated to act by the removal of the transcription factor Tcf-4 from the osteopontin promoter, where it acts normally to suppress osteopontin transcription (El-Tanani *et al*, 2001). For Tcf-4 to be removed from the osteopontin promoter by the C9-Met-DNA, the integrated copies of C9-Met-DNA in the chromatin need to be in a conformation that is accessible to proteins, that is unwound. This state would probably depend on their becoming integrated into a region of the genome of a Rama 37 cell that is actively transcribed. Different sites of integration of the concatamers of C9-Met-DNA (Chen *et al*, 1997) could explain the observation in this report that occasionally large numbers of integrated C9-Met-DNA fragments occur in transfected cells with low levels of osteopontin mRNA and a low metastatic potential (e.g. R37C9VM8).

To conclude, this report proves that the original pool of C9-Met-DNA-transfected Rama 37 cells is a mixed population of cells with various molecular and cellular characteristics. Moreover, the close association between osteopontin mRNA levels and metastatic potential of the C9-Met-DNA clonal cell lines provides further evidence that the direct effect of C9-Met-DNA is indeed mediated through the increase in osteopontin mRNA levels. Finally the pairwise linear correlations between metastatic potential, and both osteopontin mRNA levels and plating efficiency of the cells suggests that the mechanism by which osteopontin increases metastatic potential is mediated, at least in part, by increasing the adhesion of the tumour cells in this system.
